# MRI bone oedema scores are higher in the arthritis mutilans form of psoriatic arthritis and correlate with high radiographic scores for joint damage

**DOI:** 10.1186/ar2586

**Published:** 2009-01-06

**Authors:** Yu M Tan, Mikkel Østergaard, Anthony Doyle, Nicola Dalbeth, Maria Lobo, Quentin Reeves, Elizabeth Robinson, William J Taylor, Peter B Jones, Karen Pui, Jamie Lee, Fiona M McQueen

**Affiliations:** 1 Department of Molecular Medicine and Pathology, University of Auckland, Park Road, Auckland 1010, New Zealand; 2 Department of Rheumatology, Auckland District Health Board, Greenlane West, Auckland 1051, New Zealand; 3 Department of Rheumatology, Copenhagen University Hospitals at Hvidovre and Gentofte, Kettegård alle 30, Hvidovre, DK-2650, Denmark; 4 Department of Radiology, Auckland City Hospital, Grafton Rd, Auckland 1010, New Zealand; 5 Department of Epidemiology and Biostatistics, University of Auckland, Morrin Road, Auckland 92019, New Zealand; 6 Department of Medicine, University of Otago Wellington, Mein St, Wellington 6021, New Zealand; 7 Department of Rheumatology, QE Health, Whakaue St, Rotorua 3010, New Zealand

## Abstract

**Introduction:**

The aim of this study was to investigate the magnetic resonance imaging (MRI) features of bone disease in the arthritis mutilans (AM) form of psoriatic arthritis (PsA).

**Methods:**

Twenty-eight patients with erosive PsA were enrolled (median disease duration of 14 years). Using x-rays of both hands and feet, 11 patients were classified as AM and 17 as non-AM (erosive psoriatic arthritis without bone lysis)by two observers. MRI scans (1.5T) of the dominant hand (wrist and fingers scanned separately) were obtained using standard contrast-enhanced T1-weighted and fat-saturated T2-weighted sequences. Scans were scored separately by two readers for bone erosion, oedema and proliferation using a PsA MRI scoring system. X-rays were scored for erosions and joint space narrowing.

**Results:**

On MRI, 1013 bones were scored by both readers. Reliability for scoring erosions and bone oedema was high (intraclass correlation coefficients = 0.80 and 0.77 respectively) but only fair for bone proliferation (intraclass correlation coefficient = 0.42). MRI erosion scores were higher in AM patients (53.0 versus 15.0, p = 0.004) as were bone oedema and proliferation scores (14.7 versus 10.0, p = 0.056 and 3.6 versus 0.7, p = 0.003 respectively). MRI bone oedema scores correlated with MRI erosion scores and X-ray erosion and joint space narrowing scores (r = 0.65, p = 0.0002 for all) but not the disease activity score 28-C reactive protein (DAS_28CRP_) or pain scores.

**Conclusions:**

In this patient group with PsA, MRI bone oedema, erosion and proliferation were all more severe in the AM-form. Bone oedema scores did not correlate with disease activity measures but were closely associated with X-ray joint damage scores. These results suggest that MRI bone oedema may be a pre-erosive feature and that bone damage may not be coupled with joint inflammation in PsA.

## Introduction

Arthritis mutilans (AM) is the most severe and destructive of the five clinical presentations of psoriatic arthritis (PsA) as defined by Moll and Wright [1]. It is characterised by severe radiographic erosion with bony osteolysis, often resulting in digital shortening and the '*main en lorgnette*' (opera-glass hand) deformity [2]. Bone proliferation and arthrodesis may coexist with erosion in PsA and both forms of bone disease have been described in AM [3]. Magnetic resonance imaging (MRI) can reveal more information about bone pathology in inflammatory arthritis than conventional radiography (XR) as it is a multiplanar technique with the capacity to depict bone erosion and proliferation using three-dimensional imaging. MRI is the only imaging modality capable of revealing bone oedema, which in rheumatoid arthritis (RA) has been shown to be a pre-erosive change and associated with osteitis [4-6]. MRI bone oedema has also been described in PsA [7-10] where it may be diaphyseal as well as subchondral [8] and is responsive to anti-tumour necrosis factor (TNF) therapy [10]. In this study we investigated the characteristics of bone disease in erosive PsA using XR, contrast-enhanced MRI scanning and dual energy X-Ray absorptiometry (DEXA). We sought to determine whether the AM form differs from non-AM (erosive psoriatic arthritis without bone lysis) PsA using these modalities, specifically concentrating on MRI bone oedema in view of its potential role in the genesis of bone erosion.

## Materials and methods

### Patients and clinical assessments

With the approval of the New Zealand Multiregion Ethics Committee, 28 patients with PsA (as defined by Vasey and Espinzoa modified by Taylor and colleagues [11]) were recruited from Auckland, Rotorua and Wellington in New Zealand from 2005 to 2007. These patients were enrolled as part of a longitudinal study investigating the effects of zoledronic acid on the progression of bone erosions in PsA (the zoledronic acid in psoriatic arthritis or ZAPA study), but results presented here pertain only to baseline findings in these patients, before administration of the study drug or placebo. All patients gave informed consent according to the requirements of the New Zealand Multiregion Ethics Committee.

Enrolment criteria included the presence of peripheral erosions on XR confirmed by a radiologist. A total of 17 males and 11 females were enrolled and all underwent clinical assessments including collection of demographic data, as well as disease activity scores (DAS) obtained from history, examination and laboratory investigations including duration of early morning stiffness, swollen (n = 76) and tender (n = 78) joint counts, visual analogue scores for pain and overall well-being, patient and physician global assessments, erythrocyte sedimentation rate (ESR) and C-reactive protein (CRP). DAS-28_CRP _(four variable) and DAS-28_ESR _(four variable) scores were computed to indicate overall disease activity [12]. Assessments of functional disability were also obtained using the Health Assessment Questionnaire (HAQ) score [13], which has been used to assess functional limitations in PsA [14] and the Physical Function component of the Short form-36 (PF-SF-36) score [15]. Severity of psoriasis was assessed using the Psoriasis Area and Severity Index (PASI) [16] and the Psoriasis Nail Severity Score (PNSS) [17] was also used.

### Radiography

Plain XRs of the hands, feet and sacroiliac joints were obtained at enrolment. XRs were scored by a radiologist and a rheumatologist (QR and ND) for erosions and joint space narrowing according to the Sharp van der Heijde score modified for use in PsA [18]. Sacroiliitis was scored as present or absent by another clinical radiologist.

### Radiographic definition of arthritis mutilans

Patients were categorised as having AM or non-AM PsA on the basis of XR features in the peripheral joints, using the definition from Marsal and colleagues [19], which requires complete erosion of bone on both sides of the joint(s). This was performed by two readers (WT and QR) who reviewed digitised films separately and, where there was disagreement by consensus, blinded to clinical and MRI findings.

### Clinical definition of arthritis mutilans

Clinical digitised photographs of the hands and feet were obtained in 25 of the 28 patients. These were examined by a rheumatologist (ND) blinded to the results of radiography and MRI. Patients were classified as AM or non-AM according to the presence of digital shortening in the fingers or toes. Patients were also classified separately by their referring physicians as AM or non-AM.

### MRI scans

MRI scans of the wrist (distal radius and ulna, carpal bones and metacarpal bases 2 to 5) and fingers (metacarpals proximal to bases, metacarpophalangeal (MCP) joints, proximal phalanges, proximal interphalangeal (PIP) joints, middle phalanges, distal interphalangeal (DIP) joints, distal phalanges) of the dominant hand were obtained using a Siemens Magnetom Avanto 1.5 Tesla (T) scanner (Siemens, Penrose, Auckland New Zealand) with a dedicated wrist coil (small field of view at 11 cm for optimal signal-to-noise ratio). Details of sequences and acquisitions are shown in Table 1. The sequence of imaging was as follows: unenhanced imaging of the fingers; the patient was repositioned so that the wrist was within the coil; unenhanced imaging of the wrist; contrast injection; enhanced imaging of the wrist; the patient was repositioned so that the fingers were within the coil; and then enhanced imaging of the fingers was performed. Bone oedema was investigated using short tau inversion recovery (STIR) sequences, whereas bone erosion and bone proliferation were assessed on axial and coronal T1-weighted sequences. For all parameters a water-excitation volumetric interpolated breath-hold examination (3D VIBE) sequence (a gradient echo 3D T1-weighted sequence) was used as a supplement. This sequence was obtained after intravenous administration of the contrast agent, gadolinium diethylenetriamine pentaacetic acid (Gd-DTPA).

**Table 1:** MRI sequences and acquisitions

**WRIST**	FOV	SLICE	TR	TE	MATRIX
AXIAL T1	110 mm	3.0 mm	473 ms	19 ms	192 × 320
AXIAL STIR	110 mm	3.0 mm	4500 ms	59 ms	192 × 256
CORONAL T1	110 mm	3.0 mm	453 ms	19 ms	224 × 320
CORONAL STIR	110 mm	3.0 mm	4600 ms	62 ms	192 × 256
VIBE (post-contrast)	110 mm	0.6 mm	16.4 ms	6.83 ms	192 × 192
					
**FINGERS**					

CORONAL T1	110 mm	3.0 mm	453 ms	19 ms	224 × 320
AXIAL T1	110 mm	3.0 mm	633 ms	19 ms	230 × 320
SAGITTAL STIR	110 mm	3.0 mm	3140 ms	54 ms	192 × 256
VIBE (post-contrast)	110 mm	0.6 mm	16.4 ms	6.83 ms	192 × 192

FOV = field of view, STIR = short tau inversion recovery, T1 = T1-weighted, TR = repetition time, TE = echo time, VIBE = volumetric interpolated breath-hold examination.

Scans were scored separately by two trained readers (MØ and AD) for bone erosion and bone oedema using Rheumatoid Arthritis Magnetic Resonance Imaging Scoring system (RAMRIS) [20] criteria modified for PsA (Psoriatic Arthritis Magnetic Resonance Imaging Scoring System, PsAMRIS) [21]. The following bones were scored for erosion (0 to 10) and bone oedema (0 to 3): hamate, capitate, trapezoid, trapezium, triquetrum, pisiform, lunate, scaphoid, distal ulna, distal radius, bases of metacarpals (2 to 5), MCP joint region (2 to 5 proximal and distal to the joint), PIP joint region (2 to 5 proximal and distal to the joint) and DIP joint region, (2 to 5 proximal and distal to the joint). Bone proliferation was also scored at each bone site as present or absent (0 or 1). Scores were averaged across readers to provide one data set for this analysis. Data from the fingers were also analysed on the basis of individual MCP, PIP and DIP joints. A mean score for both readers was obtained at each joint for erosions, bone oedema and bone proliferation: erosions were scored (0 to 20), bone oedema (0 to 6) and bone proliferation (0 to 2) to include bone involvement on each side of the joint.

### Bone densitometry

Bone densitometry was performed at L1 to L4 and at the femoral neck using a Lunar Expert dual energy absorptiometer (GE Lunar, Madison, WI). Results were expressed as T scores representing the number of standard deviations below the average for a young adult at peak bone density. For the purposes of this analysis T scores for L1 to L4 were averaged.

### Statistical analysis

Intraclass correlation coefficients (ICC) with 95% confidence intervals (CI) were used to assess the interobserver reliability of scoring of XR and MRI features. Mann Whitney U tests and Chi squared tests were used to test differences between AM and non-AM groups in terms of demographics, disease activity, XR measures and MRI measures. Medians with ranges or interquartile ranges and percentages were used to describe these differences. Spearman's correlations were used to assess the association between MRI bone oedema scores and other measures.

## Results

**Figure 1:**
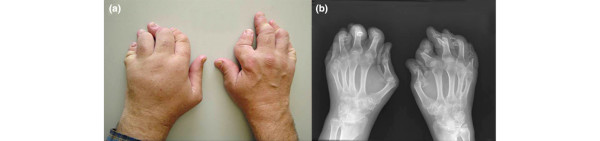
**A patient with arthritis mutilans with digital shortening**. (a) Clinical photograph. (b) Radiograph of the hands.

In total, 11 of the 28 patients were classified by the XR definition as AM and 17 as non-AM. In six cases, opinions of the XR readers differed and these were re-examined and a consensus reached. Of the 11 patients with XR-AM, seven fitted the clinical definition of AM with digital shortening (Figure 1). The following analysis has used the XR definition of AM. Table 2 shows demographic details for the AM group compared with the non-AM group, as well as their medications, DAS and functional measures.

**Table 2:** Demographics, medications and disease activity in AM and non-AM patients

**Clinical features**	AM*(N = 11)	**Non AM (N = 17)**	**p value**
		
	Median (range)	Median (range)	
Age (years)	52 (36 to 63)	50 (20 to 63)	0.56
Duration of PsA (years)	12 (5 to 35)	10 (5 to 25)	0.51
Duration of psoriasis (years)	22 (11 to 49)	20 (5 to 50)	0.24
Weight (kg) mean (range)	78 (65 to 107)	83 (68 to 111)	0.42
			
Female:Male	3:8	8:9	0.44
Ethnicity: European	91%	88%	0.94
			
**Medications**	Number (%)	Number (%)	

Methotrexate	4 (36%)	11 (65%)	
NSAIDs	7 (64%)	10 (10%)	
Prednisone 5 to 20 mg/day	2 (18%)	2 (12%)	
Sulphasalazine 2 to 3 g/day	3 (28%)	5 (29%)	
Azathioprine 150 mg/day	0	1 (6%)	
Hydroxychloroquine 400 mg/day	0	1 (6%)	
Leflunomide 20 mg/day	1 (9%)	0	
Cyclosporin 100 mg/day	0	1 (6%)	
			
**Disease activity**	Median (range)	Median (range)	

Tender joint count	17 (1 to 40)	11 (4 to 51)	0.98
Swollen joint count	6 (0 to 33)	4 (0 to 9)	0.20
Pain score	35 (16 to 78)	45 (6 to 82)	0.47
HAQ score (n = 27)	1.1 (0 to 3.5)	0.7 (0 to 3)	0.26
PF-SF-36	52.5 (5 to 85)	65 (10 to 90)	0.39
ESR (mm/hour)	14 (1 to 43)	13 (2 to 86)	0.61
CRP (mg/litre) (n = 25)	11.6 (3 to 59)	4.9 (< 1 to 46)	0.26
DAS28-CRP (n = 23)	3.91 (2.6 to 5.7)	4.2 (2.3 to 6.2)	0.64
DAS28-ESR (n = 28)	4.2 (1.7 to 6.1)	4.0 (1.9 to 6.9)	0.61
Psoriatic nail severity score	11 (0 to 47)	8 (0 to 22)	0.19
PASI (n = 26)	0.6 (0 to 12)	1.8 (0 to 10.3)	0.84

AM = arthritis mutilans, CRP = c-reactive protein, DAS28 – CRP = Disease Activity Score (28 swollen and tender joints, CRP, General Health VAS), DAS28 – ESR = Disease Activity Score (28 swollen and tender joints, ESR, General Health VAS), ESR = Erythrocyte Sedimentation Rate, HAQ = Health Assessment Questionnaire, non-AM = non-arthritis mutilans, NSAID = nonsteroidal anti-inflammatory drugs, PASI = Psoriasis Area and Severity Index, PF-SF-36 = Physical Function component of the Short Form 36 Questionnaire, PsA = psoriatic arthritis.

### Interobserver reliability for scoring XR and MRI features

XR features of erosion and joint space narrowing were assessed at the hands and feet by two observers (ND and QR). Interobserver reliability was high for each with ICCs and 95% confidence intervals (CI) as follows: erosions 0.79 (0.42 to 0.83), joint space narrowing 0.90 (0.80 to 0.95) and when combined for a modified total Sharp score (including DIP joints) 0.86 (0.74 to 0.93).

For the MRI analysis, a total of 1013 bones at the dominant wrist and fingers were scored for bone erosion, oedema and proliferation by two readers (MØ and AD) working separately in two different institutions. Reliability for scoring MRI erosions and bone oedema was high: 0.80 (0.62 to 0.90) and 0.77 (0.57 to 0.88) respectively. It was lower for bone proliferation: 0.42 (0.07 to 0.67).

### Clinical disease activity in AM versus non-AM patients

There was no difference between AM and non-AM groups in terms of DAS with respect to inflammatory markers (ESR and CRP), clinical evidence of joint inflammation (pain score, tender and swollen joints counts), joint function (HAQ score and PF-SF-36) or indicators of the severity of skin and nail disease (PASI and nail severity score) (Table 2).

### MRI and XR scores in AM vs non-AM patients

**Figure 2:**
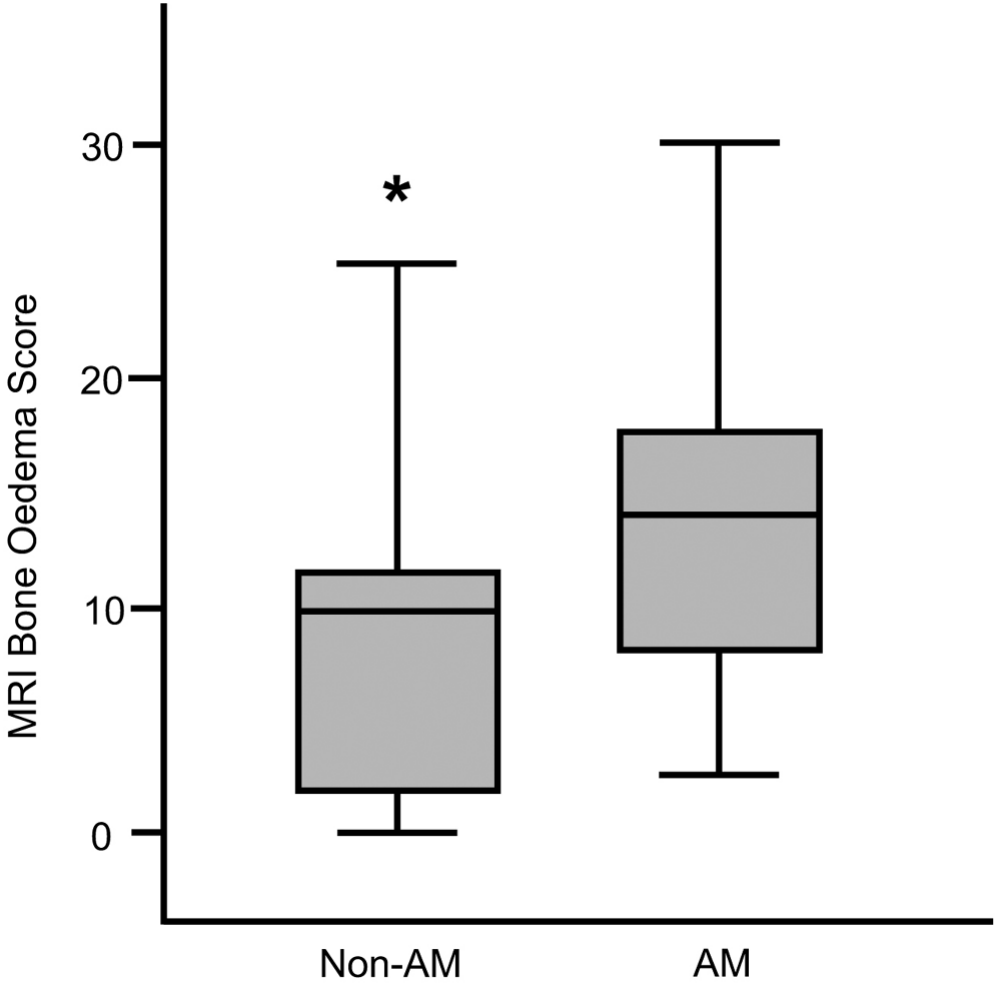
**Boxplots showing MRI bone oedema scores that are higher in AM compared with non-AM patients**. AM = arthritis mutilans, non-AM = erosive psoriatic arthritis without bone lysis, MRI = magnetic resonance imaging.

MRI scans of the dominant fingers (including DIP joints) and wrist were obtained in all patients. Table 3 summarises the data for the AM group versus the non-AM group. As expected, XR and MRI erosion scores (median) were higher in the AM group (89.8 versus 21.0, p = 0.001 and 53.0 versus 15.0, p = 0.004, respectively). When the analysis was performed on a joint-by-joint basis at the fingers, AM patients were found to have higher scores for erosions and bone proliferation (Table 3). MRI bone oedema scores were also higher in the AM group (14.7 versus 10.0, p = 0.056) (Figure 2) as were bone proliferation scores (3.6 versus 0.7, p = 0.003). Of the 304 bones where erosions were scored, 131 (43.1%) also scored positive for bone oedema. There was no difference between AM and non-AM groups in the frequency of sacroiliitis or T scores from bone densitometry (lumbar spine or hip).

**Table 3:** MRI, XR and bone densitometry in AM vs non-AM erosive PsA

**MRI (dominant wrist and fingers)**	**AM**	**non-AM**	**p value**
Bone erosion score (PAMRIS)	53.0* (28 to 125)	15.0 (3 to 22)	0.004
MCPs bone erosion**	1.8 (0.4 to 16.3)	1.0 (0 to 4)	0.045
PIPs bone erosion	2.8 (0 to 20)	0.4 (0 to 3.4)	0.036
DIPs bone erosion	1.3 (0 to 8.8)	0.0 (0 to 3.4)	0.018
Bone oedema score (PAMRIS)	14.7 (8.3 to 19.5)	10.0 (2.0 to 12.5)	0.056
MCPs bone oedema	0.0 (0 to 3)	0.0 (0 to 2.3)	0.71
PIPs bone oedema	0.0 (0 to 3.8)	0.0 (0 to 1.5)	0.74
DIPs bone oedema	0.0 (0 to 4)	0.0 (0 to 2.8)	0.74
Bone proliferation score (PAMRIS)	3.6 (2.2 to 5.0)	0.7 (0.2 to 2.1)	0.003
MCPs bone proliferation	0.3 (0 to 1)	0.0 (0 to 0.8)	0.037
PIPs bone proliferation	0.3 (0 to 0.9)	0.0 (0 to 0.5)	0.13
DIPs bone proliferation	0.3 (0 to 0.6)	0.0 (0 to 1.0)	0.021
			
**XR of hands and feet**			

XR erosion score	89.8 (69.0 to 104.3)	21.0 (6.0 to 35.0)	0.001
XR narrowing	5.0 (57.0 to 108.3)	16.5 (4.5 to 28.0)	0.002
Sacroiliitis (No. %)	3 (27%)	6 (35%)	0.98
			
**Bone densitometry**			

T score L1 -4	0.7 (- 0.1 to 4.8)	-0.1 (-1.6 to 2.8)	0.13
T score total femur	-0.4 (-1.8 to 1.3)	-0.3 (-2.1 to 1.3)	0.88

*Median + interquartile range shown

**score per joint; median (range)

AM = arthritis mutilans; DIP = distal interphalangeal; MCP = metacarpophalangeal; MRI = magnetic resonance imaging; non-AM = erosive psoriatic arthritis without bone lysis; PAMRIS = Psoriatic arthritis MRI scoring system; PIP = proximal interphalangeal; PsA = psoriatic arthritis; XR = plain radiography.

### Correlations between MRI, XR and clinical scores

**Figure 3:**
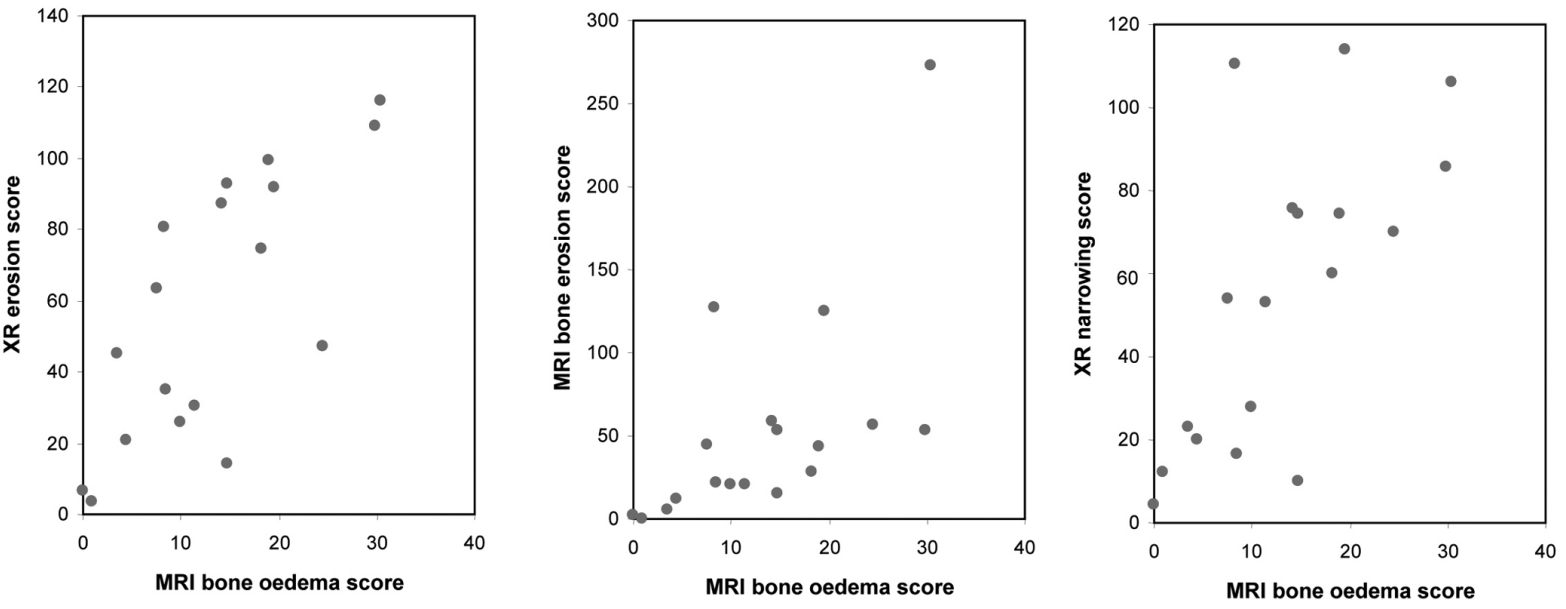
**Scatter plots showing correlations**. Correlation seen between (a) magnetic resonance imaging (MRI) bone oedema score and plain radiography (XR) erosion score (r = 0.65, p = 0.0002); (b) MRI bone oedema and MRI erosion score (r = 0.66, p = 0.0002); and (c) MRI bone oedema score and XR joint space narrowing score (r = 0.65, p = 0.0002).

**Figure 4:**
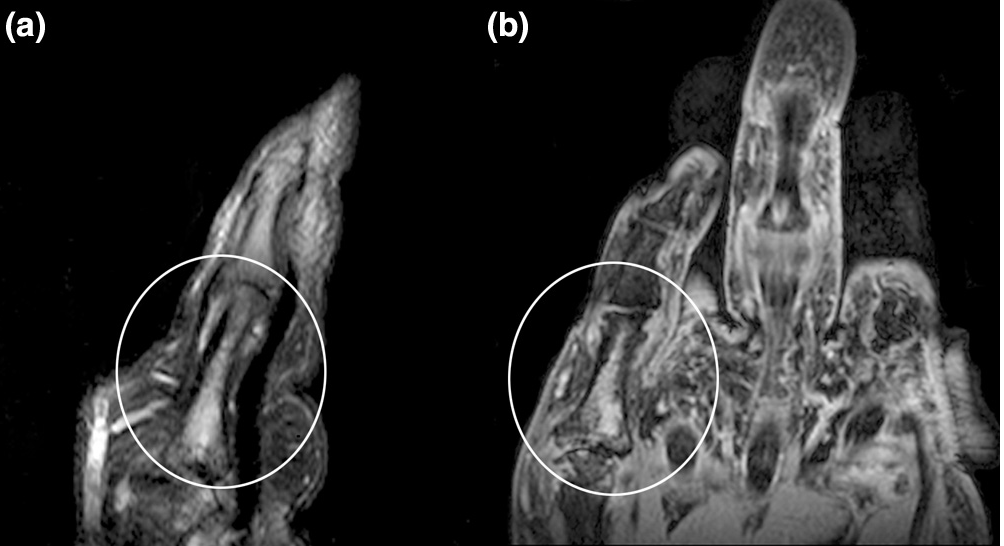
**Sagittal T2 weighted fat-saturated (FS) magnetic resonance imaging (MRI) scan of the fifth finger of a patient with non-AM**. (a) Diaphyseal bone oedema is shown (circle) and confirmed on (b) coronal post-contrast volumetric interpolated breath-hold examination (VIBE) sequence (circle).

The MRI erosion and bone oedema scores correlated strongly with the XR erosion score (r = 0.709, p < 0.0001 and r = 0.65, p = 0.0002, respectively). The MRI bone oedema score also correlated strongly with the MRI erosion score (r = 0.66, p = 0.0002) and XR total joint space narrowing score (r = 0.65, p = 0.0002) (Figure 3). Interestingly, the MRI bone oedema score did not correlate with clinical indicators of disease activity such as the DAS_28CRP _or pain scores (r = 0.18, p = 0.39 and r = 0.03, p = 0.87, respectively). Both readers scored diaphyseal bone oedema as present in six bones in four patients (one AM and three non-AM). An example is shown in Figure 4 where diaphyseal bone oedema was revealed on both STIR and VIBE sequences.

## Discussion

The MRI features of PsA have only recently begun to be explored [22]. This disease differs radiographically from RA in that bone erosion and bone proliferation are both recognised (and sometimes coexist in the same joint), although the characteristic features of spondyloarthropathies (SpA), such as sacroiliiitis and enthesitis, may also occur [23]. MRI reflects these findings and provides additional information through its capacity to image synovitis, tenosynovitis, dactylitis and also bone oedema, which has been described at subchondral, entheseal and diaphyseal locations [7]. AM represents the most severe end of the spectrum as far as bone disease is concerned in PsA with extreme bony lysis and 'pencil-in-cup' deformities resulting in digital shortening and the *main en lorgnette *deformity. In this study we have investigated bone disease in patients with AM and non-AM forms of erosive PsA using three imaging modalities; contrast-enhanced MRI, XR and DEXA. We defined AM in two ways using information from several sources and chose to use the radiographic definition of Marsal and colleagues [19] as verified by two observers. Our first concern was that this did not completely coincide with the clinical definition from digital photographs, which were assessed separately. On further investigation it became apparent that those patients fitting the clinical definition formed a subset of those defined radiographically.

For the purposes of this study we used the Psoriatic Arthritis Magnetic Resonance Imaging Scoring system (PsAMRIS) currently being developed and validated by an ongoing Outcome Measures in Rheumatology Clinical Trials (OMERACT)-based project [21]. This involved scoring bone erosion, oedema and proliferation at the sites dictated by the RAMRIS system [20] with the addition of the PIP and DIP joints. These data were obtained from review of a very large number of bony regions (1013) by two readers working completely independently in different institutions. A high degree of inter-reader reliability was demonstrated both for bone erosions and bone oedema (ICCs of 0.8 and 0.77, respectively), despite the fact that many patients had extremely advanced and deforming disease, making many regions difficult to assess. Bone proliferation data are also presented although the interobserver reliability was only moderate (ICC = 0.42), possibly because of the difficulty in recognising proliferation when it appears adjacent to regions of severe erosion. In another group of PsA patients with relatively early disease, the ICC for the bone proliferation component of PsAMRIS was much higher at 0.91 (unpublished data) and this emphasises the heterogeneity of PsA and the fact that this system for scoring disease features may perform differently in different patient groups.

As expected, the AM group had higher XR erosion and joint space narrowing scores at the hands and feet than non-AM patients and this was also true for MRI erosions at the dominant fingers and wrist. A major new finding was that MRI bone oedema was also higher in the AM group. Interestingly, bone oedema scores were highly correlated with MRI and XR erosion and joint space narrowing scores, suggesting that this feature occurs in those with more severe, damaging bone disease. We did not find an association with functional scores, pain or disease activity and this is consistent with observations in other SpA [24,25] but differs from findings in RA, where there is good evidence that bone oedema is an inflammatory indicator that correlates with CRP in early and established disease [4,26]. Clinical studies have also suggested that RA and PsA differ in terms of the CRP and other markers of disease activity [14,27]. Buskila and coleagues noted that PsA patients reported less tenderness of inflamed joints than RA patients and concluded that the DAS28 may not adequately reflect the burden of inflammation in PsA for this reason and also because it excludes the DIP and foot joints [28].

This study has revealed a number of negative findings. We did not find a particular association between AM and sacroiliitis as has been noted previously [19]. This is probably because we enrolled a relatively homogeneous group of patients with erosive PsA only, whereas studies that have found sacroiliitis to be more common in the AM form have used a broader group of PsA patients with erosive and non-erosive disease as their denominator. Another negative finding from this study was that bone density measurements at the femoral neck and lumbar spine did not differ between the AM and non-AM groups. In RA, those patients with the most active, erosive disease tend to be those with the most severe osteopenia, both periarticular and generalised [29]. Periarticular osteopenia is not a feature of PsA [30] but one study has shown that bone mineral density at the spine in PsA patients is lower than normal controls [31]. Grisar and colleagues found evidence that markers of bone resorption were increased in PsA patients and correlated with the acute phase response [32], but they did not examine the association between BMD and CRP which was not significant in our group.

## Conclusion

To the best of our knowledge, we have presented the first MRI study investigating the AM variant of PsA. We confirmed that MRI and XR joint damage (erosion) and proliferation scores were higher in the AM group than in those with non-AM erosive PsA, despite there being no evidence of greater disease activity in terms of clinical scores (skin or joint) or inflammatory markers. Interestingly, the MRI bone oedema score was also higher in the AM group and correlated strongly with erosion and joint space narrowing scores. These data suggest that MRI bone oedema could be a forerunner of articular damage in PsA and may be a useful biomarker to indicate aggressive disease. Follow-up of this group is planned to explore the evolution of these changes over time.

## Abbreviations

AM: arthritis mutilans; CI: confidence interval; CRP: C-reactive protein; DAS: disease activity score; DEXA: dual energy XRay absorptiometry; DIP: distal interphalangeal; ESR: erythrocyte sedimentation rate; Gd-DTPA: gadolinium diethylenetriamine pentaacetic acid; HAQ: Health Assessment Questionnaire; MCP: metacarpophalangeal; MRI: magnetic resonance imaging; non-AM: erosive psoriatic arthritis without bone lysis; OMERACT: Outcome Measures in Rheumatology Clinical Trials; PAMRIS: Psoriatic arthritis MRI scoring system; PASI: Psoriasis Area and Severity Index; PF-SF-36: Physical Function component of the Short form-36; PIP: proximal interphalangeal; PNSS: Psoriasis Nail Severity Score; PsA: psoriatic arthritis; RA: rheumatoid arthritis; RAMRIS: Rheumatoid Arthritis Magnetic Resonance Imaging Scoring system; PsAMRIS: Psoriatic Arthritis Magnetic Resonance Imaging Scoring system; SpA: spondyloarthropathies; STIR: short tau inversion recovery; TNF: tumour necrosis factor; 3D VIBE: three-dimensional volumetric interpolated breath-hold examination; XR: plain radiography.

## Competing interests

The authors declare that they have no competing interests.

## Authors' contributions

YMT carried out data analysis, and assisted in manuscript preparation. MØ participated in the design of the study, was a reader for the MRI scans and assisted in manuscript preparation. AD participated in the design of the study, was a reader for the MRI scans and assisted in manuscript preparation. ND assisted in patient recruitment, was a reader for the X-rays and assisted in manuscript preparation. ML assisted in patient recruitment and participated in data analysis. QR was a reader for the X-rays and assisted in manuscript preparation. ER provided statistical advice and assisted in data analysis and manuscript preparation. WJT assisted in patient recruitment and manuscript preparation. PBJ participated in the design of the study and assisted in patient recruitment. KP assisted in patient recruitment and participated in data entry. JL participated in data entry and analysis. FMM conceived of the study and coordinated patient recruitment, data entry, data analysis and preparation of the manuscript.
